# Abnormal pattern of brain glucose metabolism in Parkinson’s disease: replication in three European cohorts

**DOI:** 10.1007/s00259-019-04570-7

**Published:** 2019-11-25

**Authors:** Sanne K. Meles, Remco J. Renken, Marco Pagani, L. K. Teune, Dario Arnaldi, Silvia Morbelli, Flavio Nobili, Teus van Laar, Jose A. Obeso, Maria C. Rodríguez-Oroz, Klaus L. Leenders

**Affiliations:** 1 Department of Neurology, University of Groningen, University Medical Center Groningen, Groningen, The Netherlands; 2 Neuroimaging Center, Department of Neuroscience, University of Groningen, Groningen, The Netherlands; 3 Institutes of Cognitive Sciences and Technologies, CNR, Rome, Italy; 4 Department of Medical Radiation Physics and Nuclear Medicine, Karolinska University Hospital, Stockholm, Sweden; 5 Department of Nuclear Medicine, University of Groningen, University Medical Center Groningen, Groningen, The Netherlands; 6 Clinical Neurology, Department of Neuroscience (DINOGMI), University of Genoa, Genoa, Italy; 7 IRCCS Ospedale Policlinico San Martino, Genoa, Italy; 8 Nuclear Medicine, Department of Health Sciences (DISSAL), University of Genoa, Genoa, Italy; 9 Neurosciences Area, CIMA, Neurology and Neurosurgery, Clínica Universidad de Navarra, Pamplona, Spain; 10 Centro de Investigación Biomédica en Red sobre Enfermedades Neurodegenerativas (CIBERNED), Madrid, Spain; 11 CINAC, HM Puerta del Sur, Hospitales de Madrid, and Medical School, CEU-San Pablo University, Madrid, Spain; 12 Department of Neurology, Clinica Universidad de Navarra, Universidad de Navarra, Pamplona, Spain; 13 BCBL. Basque Center on Cognition, Brain and Language, Donostia-San Sebastián, Spain; 14 Ikerbasque, Basque Foundation for Science, Bilbao, Spain; 15 Present Address: Department of Neurology, Wilhelmina Ziekenhuis, Assen, Netherlands

**Keywords:** ^18^F-FDG PET, Parkinson’s disease, Metabolic pattern, Networks

## Abstract

**Rationale:**

In Parkinson’s disease (PD), spatial covariance analysis of ^18^F-FDG PET data has consistently revealed a characteristic PD-related brain pattern (PDRP). By quantifying PDRP expression on a scan-by-scan basis, this technique allows objective assessment of disease activity in individual subjects. We provide a further validation of the PDRP by applying spatial covariance analysis to PD cohorts from the Netherlands (NL), Italy (IT), and Spain (SP).

**Methods:**

The PDRP_NL_ was previously identified (17 controls, 19 PD) and its expression was determined in 19 healthy controls and 20 PD patients from the Netherlands. The PDRP_IT_ was identified in 20 controls and 20 “de-novo” PD patients from an Italian cohort. A further 24 controls and 18 “de-novo” Italian patients were used for validation. The PDRP_SP_ was identified in 19 controls and 19 PD patients from a Spanish cohort with late-stage PD. Thirty Spanish PD patients were used for validation. Patterns of the three centers were visually compared and then cross-validated. Furthermore, PDRP expression was determined in 8 patients with multiple system atrophy.

**Results:**

A PDRP could be identified in each cohort. Each PDRP was characterized by relative hypermetabolism in the thalamus, putamen/pallidum, pons, cerebellum, and motor cortex. These changes co-varied with variable degrees of hypometabolism in posterior parietal, occipital, and frontal cortices. Frontal hypometabolism was less pronounced in “de-novo” PD subjects (Italian cohort). Occipital hypometabolism was more pronounced in late-stage PD subjects (Spanish cohort). PDRP_IT_, PDRP_NL_, and PDRP_SP_ were significantly expressed in PD patients compared with controls in validation cohorts from the same center (*P* < 0.0001), and maintained significance on cross-validation (*P* < 0.005). PDRP expression was absent in MSA.

**Conclusion:**

The PDRP is a reproducible disease characteristic across PD populations and scanning platforms globally. Further study is needed to identify the topography of specific PD subtypes, and to identify and correct for center-specific effects.

**Electronic supplementary material:**

The online version of this article (10.1007/s00259-019-04570-7) contains supplementary material, which is available to authorized users.

## Introduction

Parkinson’s disease (PD) is a common neurodegenerative disorder, for which only symptomatic therapies are available. Efforts to develop neuroprotective or preventive treatments will benefit from a reliable biomarker. Ideally, such a biomarker can identify PD in its early stages, differentiate between PD and other neurodegenerative parkinsonian disorders, track disease progression, and quantify treatment effects.

In PD, abnormal accumulation of α-synuclein in neurons impairs synaptic signaling, causing disintegration of specific neural networks [1]. Neuro-imaging with [^18^F]-fluorodeoxyglucose positron emission tomography (^18^F-FDG PET) can capture synaptic dysfunction in vivo. The radiotracer ^18^F-FDG provides an index for the cerebral metabolic rate of glucose, which is strongly associated with neuronal activity and synaptic integrity [2].

Brain regions with altered ^18^F-FDG uptake in PD have been identified with univariate group comparisons using Statistical Parametric Mapping (SPM) [3–7]. However, because metabolic activity is correlated in functionally interconnected brain regions, analysis of covariance is more suitable to assess whole-brain networks. Multivariate disease-related patterns can be identified with the Scaled Subprofile Model and Principal Component Analysis (SSM PCA). Subsequently, a disease-related pattern can be used to quantify the ^18^F-FDG PET scans of new subjects [8–10]. In this procedure, an individual’s scan is projected onto the pattern, resulting in a subject score. This is a single numeric value which reflects the degree of pattern expression in that individual’s scan.

The PD-related pattern (PDRP) was initially identified by Eidelberg et al. with SSM PCA in 33 healthy controls and 33 PD patients from the USA [11]. This PDRP (PDRP_USA_) has served as a reference in many consecutive studies [12]. The PDRP_USA_ is characterized by relatively increased metabolism of the thalamus, globus pallidus/putamen, cerebellum and pons, and by relative hypometabolism of the occipital, temporal, parietal, and frontal cortices. PDRP_USA_ subject scores were significantly correlated with motor symptoms and presynaptic dopaminergic deficits in the posterior striatum [13], increased with disease progression [14], and were shown to decrease after effective treatment [15, 16]. PDRP_USA_ was significantly expressed in patients with idiopathic REM sleep behavior disorder (iRBD), a well-known prodromal disease stage of PD [17], and could discriminate between healthy controls, PD, and patients with multiple system atrophy (MSA) [18, 19].

Because of these properties, PDRP_USA_ is considered a neuro-imaging biomarker for PD [12]. It is essential that the PDRP is thoroughly validated. In collaboration with Eidelberg et al., PDRPs were identified in independent American, Indian, Chinese, and Slovenian populations [11, 15, 20, 21]. Independently from these authors, the PDRP was recently derived in an Israeli population [22]. These PDRPs were highly similar to the PDRP_USA_, although minor deviations in PDRP regional topography were observed in several of these studies, which may be caused by differences in demographics or clinical characteristics of the cohorts.

We previously identified a PDRP in a retrospective cohort of PD patients scanned on dopaminergic medication [23], and subsequently in an independent cohort of prospectively included PD patients who were in the off-state (PDRP_NL_) [24]. We used code written in-house, and obtained similar results compared with other PDRP studies. Recently, we demonstrated significant expression of the PDRP_NL_ in idiopathic REM sleep behavior disorder (a prodromal stage of PD), PD, and dementia with Lewy bodies [25]. However, the PDRP_NL_ has not been validated in a larger cohort, and correlations with PDRP_USA_ were not explored.

The aim of the current study was to validate the PDRP_NL_ in several independent cohorts. We were able to test the PDRP_NL_ in 19 controls and 20 PD patients from our own clinic in the Netherlands, in 44 healthy controls and 38 “de-novo” PD patients from Italy, and 19 healthy controls and 49 late-stage PD patients from Spain. In addition, we newly identified a PDRP in Italian and Spanish datasets and performed a cross-validation between these populations. We compared the three PDRPs to the reference pattern (PDRP_USA_).

## Methods

### ^18^F-FDG PET data from the Netherlands

The PDRP_NL_ was previously identified in ^18^F-FDG PET scans from 17 healthy controls and 19 PD patients (NL1; Table 1) [24]. In these subjects, antiparkinsonian medication was withheld for at least 12 h before PET scanning.

**Table 1:** Dutch (NL) data

	PDRP_NL_ derivation (NL1) data from [24]		PDRP_NL_ validation (NL2) data from: [25]		MSA patients
	HC	PD	HC	PD	
*N*	17	19	19	20	8
Age	61.1 ± 7.4	63.7 ± 7.5	62.4 ± 7.5	67.5 ± 8.6	65 ± 9
Gender; *n* male %	12 (71%)	13 (68%)	9 (47%)	16 (80%)	6 (75%)
H&Y stage 1 (*n*)		10		8	
H&Y stage 2 (*n*)		9		11	
H&Y stage 3 (*n*)		0		0	
H&Y stage 4 (*n*)		0		1	
Disease duration (years)		4.4 ± 3.2 (range 1.5 to 11.5 years)		4.4 ± 5.3	3.8 ± 2.3
UPDRS-III (off)		18.4 ± 7.4		NA	NA
MMSE (NL1) or MoCA (NL2)	29.4 ± 0.9	28.5 ± 1.1	28.3 ± 1.6	NA	NA
Acquisition protocol	30 min after injection of 200 MBq of ^18^F-FDG, scan acquisition time of 6 min. Eyes closed				
Camera	Siemens Biograph mCT-64				
Reconstruction	OSEM 3D, 3i24s		uHD (PSF + TOF), 3i21s		
Matrix	400 × 400		256 × 256		
Voxel size	2.00 × 2.03 × 2.03		2.00 × 3.18 × 3.18		
Smoothing	5 mm FWHM; and 1 0 mm after intensity normalization		8 mm FWHM		
Medication	Off		8 off, 12 on medication		

Values are mean and standard deviation, unless otherwise specified

*Disease duration*, approximate time from first motor symptoms until scanning; *H&Y*, Hoehn and Yahr stage; *MMSE*, mini-mental state examination; *MoCA*, Montreal Cognitive Assessment; *UPDRS-III*, part three of the Unified Parkinson’s Disease Rating Scale (2003 version); *NA*, not available

In a previous study, we demonstrated that the PDRP_NL_ was significantly expressed in an independent dataset of 20 PD patients compared with 19 controls (NL2; Table 1) [25]. For the current study, we added scans of 8 patients with the parkinsonian variant of MSA (MSA-P). Patients were diagnosed with probable PD or MSA-P by a movement disorder specialist [26]. ^18^F-FDG-PET was performed in our clinic as part of routine diagnostic workup. These patients were scanned with the same camera as NL1. However, since the PDRP_NL_ derivation study [24], reconstruction algorithms were updated (Table 1). Antiparkinsonian medication was *not* routinely withheld in NL2 PD patients.

**Table 2:** Italian (IT) data

Data from [27]						
	Total dataset		PDRP_IT_ derivation		PDRP_IT_ validation	
	HC	PD	HC	PD	HC	PD
*N*	44	38	20	20	24	18
Age	68.8 ± 9.7	71.5 ± 6.9	68.8 ± 9.7	70.5 ± 7.3	68.8 ± 10.0	72.8 ± 6.4
Gender; *n* male %	32 (73%)	25 (65.8%)	14 (70%)	11 (55%)	18 (75%)	14 (78%)
H&Y stage 1 (*n*)		23		10		13
H&Y stage 2 (*n*)		15		10		5
Non-MCI (*n*)		18		9		9
MCI (*n*)		20		11		9
PD symptom duration (months)*		19.3 ± 13.6		20.5 ± 13.3		18.4 ± 14.4
UPDRS-III (off)		15.2 ± 6.9		15.5 ± 7.3		14.9 ± 6.4
MMSE	29.1 ± 1.0	27.7 ± 2.3	28.8 ± 1.2	27.5 ± 2.9	29.4 ± 0.6	27.9 ± 1.1
Acquisition protocol	Acquisition 45 min after injection of 200 MBq of ^18^F-FDG, scan acquisition time of 15 min. Eyes closed.					
Camera	Siemens Biograph 16 PET/CT					
Reconstruction	OSEM 3D					
Matrix	128 × 128					
Voxel size	1.33 × 1.33 × 2.00 mm					
Smoothing	8 mm FWHM after intensity-normalization					
Medication	Treatment naive					

Values are mean and standard deviation, unless otherwise specified

*Disease duration*, approximate time from first motor symptoms until scanning (in months); *H&Y*, Hoehn and Yahr stage; *MMSE*, mini-mental state examination; *UPDRS-III*, part three of the Unified Parkinson’s Disease Rating Scale (2003 version); MCI, Mild Cognitive Impairment

### ^18^F-FDG PET data from Italy

The IT dataset consisted of ^18^F-FDG PET scans from 44 healthy controls and 38 consecutive outpatients with “de-novo,” drug-naïve PD [27] (Table 2). ^123^I-FP-CIT Single Photon Emission Computed Tomography (DAT SPECT) was abnormal in all Italian PD patients. Disease-related patterns are typically determined on approximately 20 patients and 20 controls. Therefore, 20 controls and 20 patients were randomly selected from the IT dataset for PDRP_IT_ derivation. The remaining 24 controls and 18 patients were used for validation.

### ^18^F-FDG PET data from Spain

^18^F-FDG PET scans from 49 PD patients and 19 controls from Spain (SP) were included from a previous study (Table 3) [28]. Patients in this cohort had long disease durations and were studied in the *on* state (i.e., antiparkinsonian medication was continued). From this dataset, 19 PD patients were randomly selected for PDRP_SP_ identification. The remaining 30 patients were used for validation.

**Table 3:** Spanish (SP) data

Data from [28]				
	Total	PDRP_SP_ derivation		PDRP_SP_ validation
	PD	HC	PD	PD
*N*	49	19	19	30
Age	69.6 ± 5.9	68.1 ± 3.2	69.2 ± 6.1	69.8 ± 5.9
Gender (*n* male)	29 (59%)	10 (53%)	13 (68%)	16 (53%)
H&Y^†^ stage 1 (*n*)	4		0	4
H&Y stage 2 (*n*)	14		6	8
H&Y stage 3 (*n*)	24		10	14
H&Y stage 4 (*n*)	5		3	2
Non-MCI (*n*)	21		11	10
MCI (*n*)	28		8	20
Disease duration	13.4 ± 5.2		14.4 ± 4.9	12.8 ± 5.3
UPDRS-III (on)	17.2 ± 8.3		17.5 ± 6.8	16.9 ± 9.1
MMSE	27.6 ± 2.3		28.5 ± 1.8	27.1 ± 2.4
Acquisition protocol	Acquisition 40 min after injection of 370 MBq of 18F-FDG, scan acquisition time of 20 min. Eyes closed.			
Camera	Siemens ECAT EXAT HR+			
Reconstruction	Filtered back-projection			
Matrix	128 × 128			
Voxel size	2.06 × 2.06 × 2.06			
Smoothing	10 mm FWHM after intensity normalization			
Medication	On state			

Values are mean and standard deviation, unless otherwise specified

*Disease duration*, approximate time from first motor symptoms until scanning; *H&Y*, Hoehn and Yahr stage; *MMSE*, mini-mental state examination; *UPDRS-III*, part three of the Unified Parkinson’s Disease Rating Scale (2003 version); MCI, Mild Cognitive Impairment

^†^For 2 patients in the SP dataset, H&Y stage was not available

### Identification of PDRP_NL_, PDRP_IT_, and PDRP_SP_

All images were spatially normalized onto an ^18^F-FDG PET template in Montreal Neurological Institute brain space [29] using SPM12 software (Wellcome Department of Imaging Neuroscience, Institute of Neurology, London, UK).

Identification of the PDRP_NL_ was described previously [24]. For identification of the PDRP_IT_ and PDRP_SP_, we applied an automated algorithm written in-house, based on the SSM PCA method of Spetsieris and Eidelberg [10], implemented in MATLAB (version 2017b; MathWorks, Natick, MA). Images were masked to remove out-of-brain voxels, log-transformed, and subject and group means were removed. Principal component analysis (PCA) was applied to the residual profiles in voxel space, and the components explaining the top 50% of the total variance were selected for further analysis. For each subject, a score was calculated on each selected principal component (PC). These scores were entered into a forward stepwise logistic regression analysis. The components that could best discriminate between controls and patients [30] were linearly combined to form the PDRP. In this linear combination, each component was weighted by the coefficient resulting from the logistic regression model. All voxel weights in the PDRP were overlaid on a T1 MRI template in Montreal Neurological Institute (MNI) space for visualization. All voxels in the PDRP are used for subject score calculation.

To investigate which regions in each PDRP were stable, a bootstrap resampling was performed within each derivation set (1000 repetitions) [31]. Voxels that survived a one-sided confidence interval (CI) threshold of 90% (percentile method) after bootstrapping were overlaid on a T1 MRI template. The stable regions in the three PDRPs were visually compared.

### Validation of PDRP_NL_, PDRP_IT_, and PDRP_SP_

For validation, subject scores for PDRP_NL_, PDRP_IT_, and PDRP_SP_ were calculated in patients and controls from the same population. First, images were log-transformed and the subject mean and group mean (originating from the PDRP identification cohort) were removed, resulting in a residual profile for each subject. The subject score is calculated by projecting the subject residual profile on the pattern. To account for differences in data-acquisition, subject scores were always *z*-transformed to the subject scores of healthy controls that were scanned on the same camera, with identical reconstruction algorithms. If subject scores in validation PD subjects were significantly higher compared with subject scores in controls, the pattern was considered valid.

In this manner, PDRP_NL_ subject scores were calculated in the derivation cohort (NL1) and in the validation cohort (NL2). However, data acquisition was not identical for NL1 and NL2 data. This resulted in a significant difference in PDRP_NL_ subject scores between the NL1 and NL2 healthy control groups (supplementary Fig 1). To correct for these differences, subject scores in NL1 were *z*-transformed to NL1 healthy controls, such that NL1 control mean was 0 with a standard deviation of 1. Similarly, subject scores in NL2 were *z*-transformed to NL2 controls.

Subject scores for PDRP_IT_ were calculated in the IT derivation cohort (controls *n* = 20; PD *n* = 20) and the IT validation cohort (controls *n* = 24; PD *n* = 18). Because all IT scans were acquired with identical protocols, subject scores could be *z*-transformed to the IT healthy controls from the derivation sample (*n* = 20).

Subject scores for the PDRP_SP_ were calculated in the SP derivation cohort (controls *n* = 19; PD *n* = 19) and the SP validation cohort (PD *n* = 30). PDRP_SP_ subject scores were *z*-transformed to the SP controls from the derivation sample (*n* = 19). As a second SP healthy control cohort was not available, PDRP_SP_ subject scores in PD patients were compared with the PDRP_SP_ subject scores in the derivation healthy controls.

### Cross-validation of PDRP_NL_, PDRP_IT_, and PDRP_SP_

Subsequently, PDRP_NL_ subject scores were determined in the IT and SP datasets, PDRP_IT_ subject scores were determined in the NL and SP datasets, and PDRP_SP_ subject scores were determined in the NL and IT datasets. In addition, subject scores for the PDRP_USA_ were calculated in each dataset in the same manner. Each subject score was then transformed into a *z*-score with respect to controls from the same camera, such that control mean was 0 with a standard deviation of 1. To determine the performance of each pattern in discriminating between controls and patients, a receiver operating curve was plotted (for each pattern in each dataset) and the area under the curve (AUC) was obtained.

The similarity of the three PDRPs to each other and to the PDRP_USA_ was tested in two ways. First, in each dataset, the *z*-scores for each PDRP were correlated with Pearson’s *r* correlation coefficient. Second, the voxelwise topographies of the different patterns were compared by using volume-of-interest (VOI) correlations over the whole brain. A set of 30 standardized VOIs were selected from a previous study [21, 32], reflecting key nodes of the reference PDRP. Within each VOI, region weights were calculated for each pattern. Subsequently, region weights between any two of the patterns were correlated using Pearson’s r correlation coefficient.

### PDRP expression in MSA-p subjects

Subject scores for each PDRP were calculated in 8 MSA-p patients. Subject scores for each PDRP were *z*-transformed to corresponding subject scores in NL2 controls.

### Principal component 1

PDRP_USA_ [11], as well as the PDRP determined in Chinese [20] and Slovenian [21] populations, consisted of PC1 in isolation. Combinations of components were not considered. There are several methods to decide which components are disease-related and should be included in the final disease-related pattern [10]. In the current study, this decision was based on a forward stepwise logistic regression model, using the Akaike information criterion (AIC) as model selection criterion [30], in order to combine the least possible number of components to obtain the optimum discrimination between controls and patients. It is possible that the optimal model selects one component. If the PDRPs identified in the current study were *not* based on PC1 in isolation, we repeated all analyses using PC1 alone for each cohort. In that case, we additionally identified PDRP_NL_-PC1, PDP_IT_-PC1, and PDRP_SP_-PC1, and repeated the cross-validation.

### Statistical procedures

Between-group differences in PDRP *z*-scores were assessed using a Student’s *t* test. Correlations between PDRP and age, disease duration, and UPDRS were examined with Pearson’s *r* correlation coefficient. Analyses were performed using SPSS software version 20 (SPSS Inc., Chicago, IL) and considered significant for *P* < 0.05 (uncorrected).

## Results

### PDRP_NL_

**Fig. 1:**
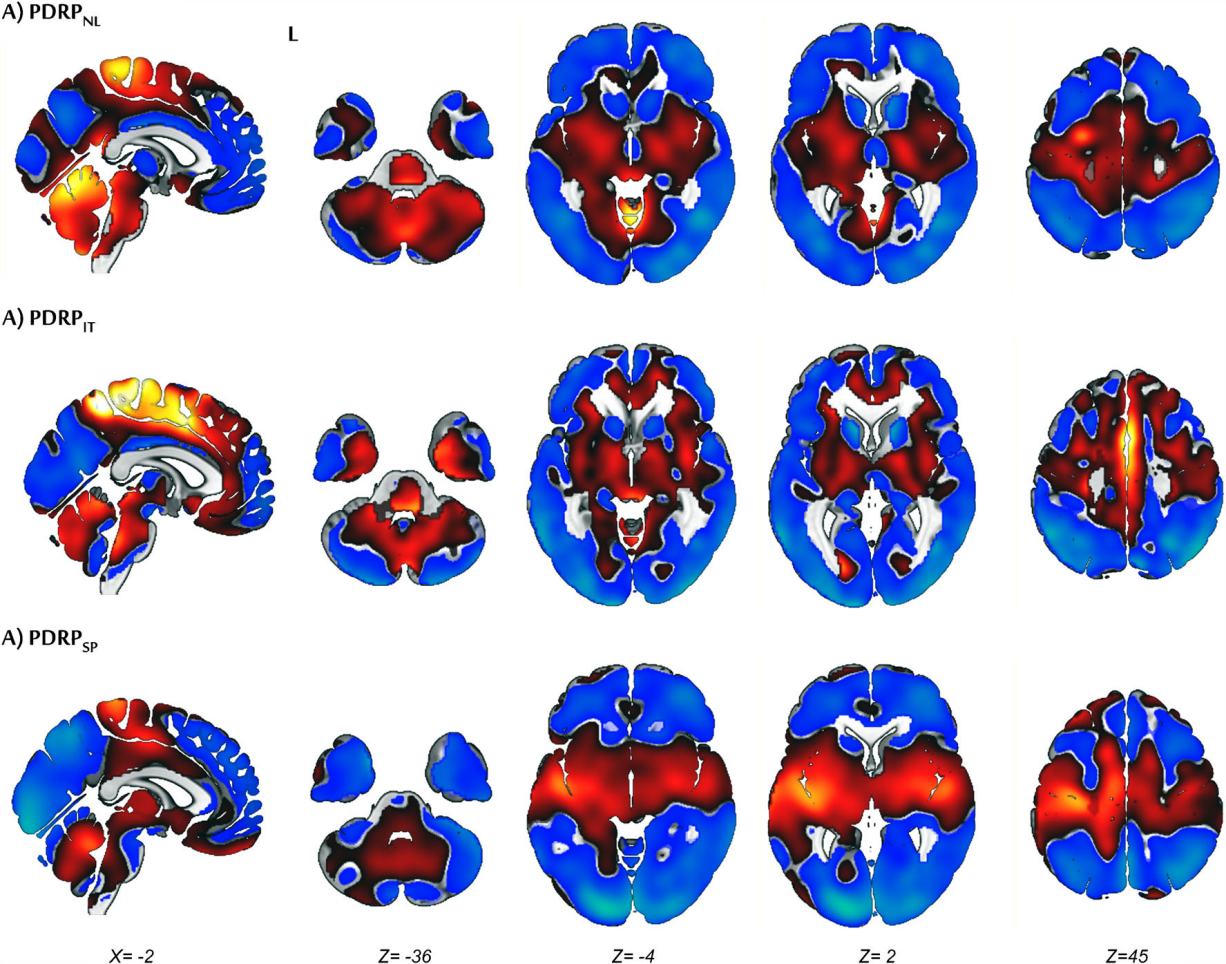
Display of PDRP_NL_ (**a**), PDRP_IT_ (**b**), and PDRP_SP_ (**c**). All voxel values of each PDRP are overlaid on a T1 MRI template. Red indicates positive voxel weights (relative hypermetabolism) and blue indicates negative voxel weights (relative hypometabolism).L=left. Coordinates in the axial (Z) and sagittal (X) planes are in Montreal Neurological Institute (MNI) standard space.

**Fig. 2:**
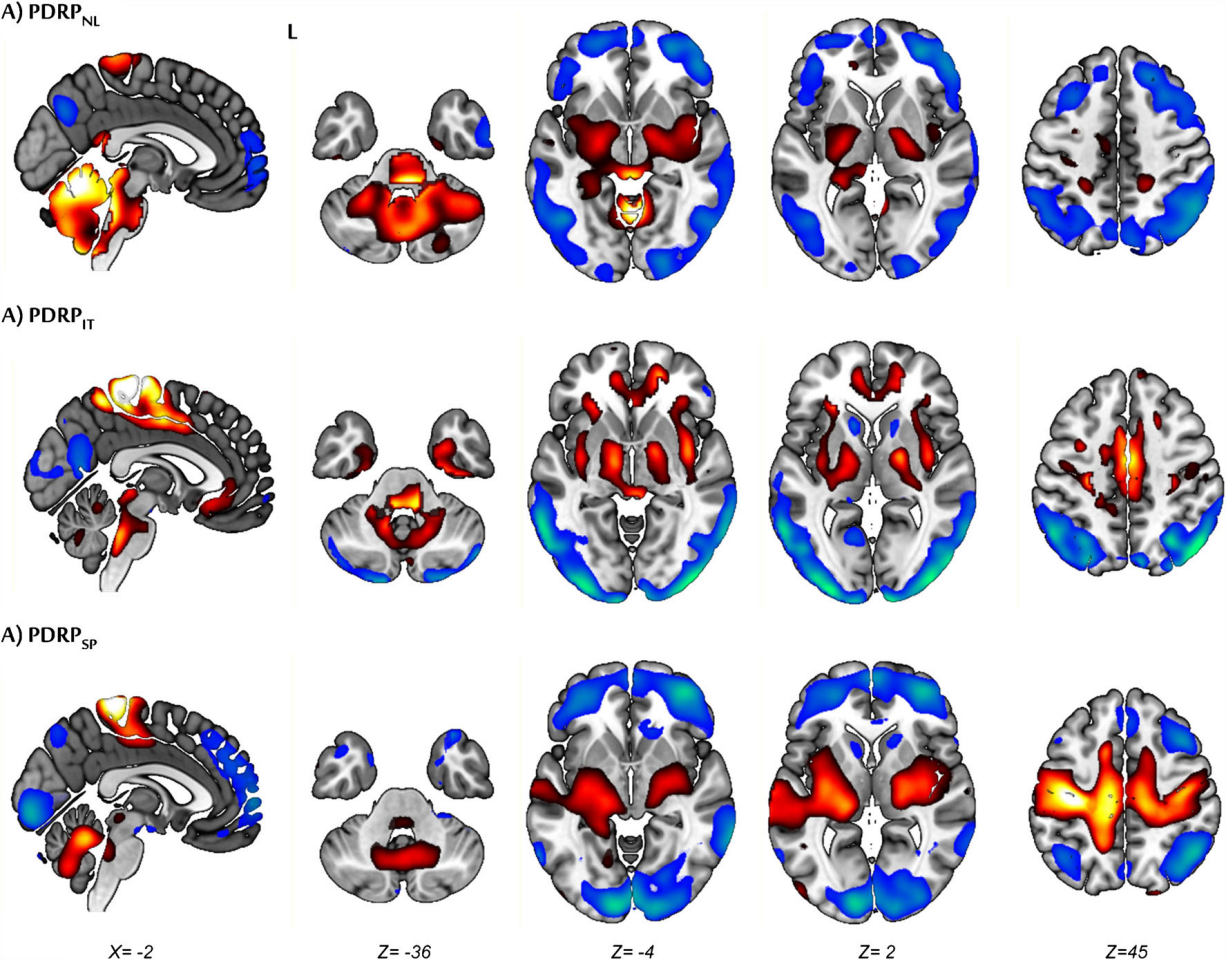
Display of stable voxels of each PDRP, determined after bootstrap resampling (90% confidence interval not straddling zero). Overlay on a T1 MRI template. Positive voxel weights are color-coded red (relative hypermetabolism), and negative voxel weights are color-coded blue (relative hypometabolism). L, left. Coordinates in the axial (Z) and sagittal (X) planes are in Montreal Neurological Institute (MNI) standard space.

**Fig. 3:**
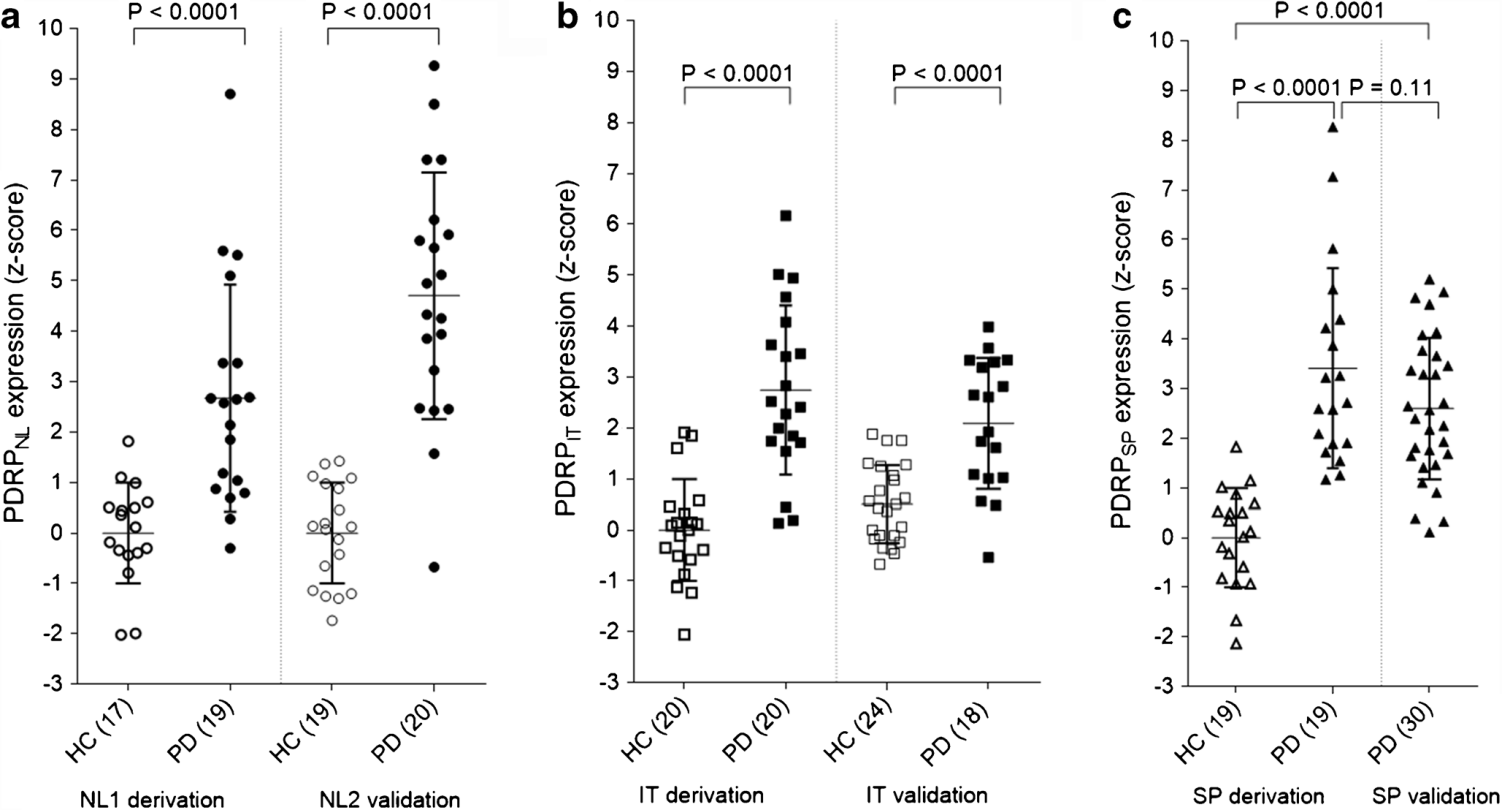
Subject scores for each PDRP in their respective derivation and validation cohorts. **a** PDRP_NL_ was identified in 17 HC and 19 PD (NL1) and validated in 19 HC and 20 PD (NL2). Because reconstruction parameters were different for cohort NL1 and NL2, PDRP subject scores were *z*-transformed to corresponding healthy controls. **b** PDRP_IT_ was identified in 20 HC and 20 PD, and validated in 24 HC and 18 PD. All subject scores were *z*-transformed to the 20 HC from the derivation sample. **c** PDRP_SP_ was identified in 19 HC and 19 PD, and validated in 30 PD. Additional HC for validation were not available. All subject scores were *z*-transformed to the 19 HC from the derivation sample. Subject *z*-scores are compared between groups with a Student’s *t* test. Bars indicate mean and standard deviation

The first six principal components explained 50% of the total variance. The PDRP_NL_ was formed by a weighted linear combination of principal components 1 and 2 (variance explained 17% and 10%, respectively; Figs. 1a and 2a). PDRP_NL_*z*-scores were significantly different between healthy controls and PD patients in both derivation (NL1) and validation (NL2) cohorts (*P* < 0.0001; Fig. 3a).

### PDRP_IT_

The first six principal components explained 51% of the total variance. A weighted linear combination of principal components 1 and 2 (variance explained 19% and 8% respectively) could best discriminate between controls and patients in the logistic regression model, and was termed the PDRP_IT_ (Figs. 1b and 2b). PDRP_IT_ subject scores were significantly different between healthy controls (*n* = 24) and patients (*n* = 18) in the validation cohort (*P* < 0.0001; Fig. 3b).

### PDRP_SP_

The first five principal components explained 51% of the total variance. The PDRP_SP_ was formed by a weighted linear combination of principal components 1, 2, and 3 (variance explained 17%, 14%, and 5%, respectively; Figs. 1c and 2c). PDRP_SP_ was significantly expressed in PD patients from the validation set (*P* < 0.0001, Fig. 3c).

### Cross-validation

**Fig. 4:**
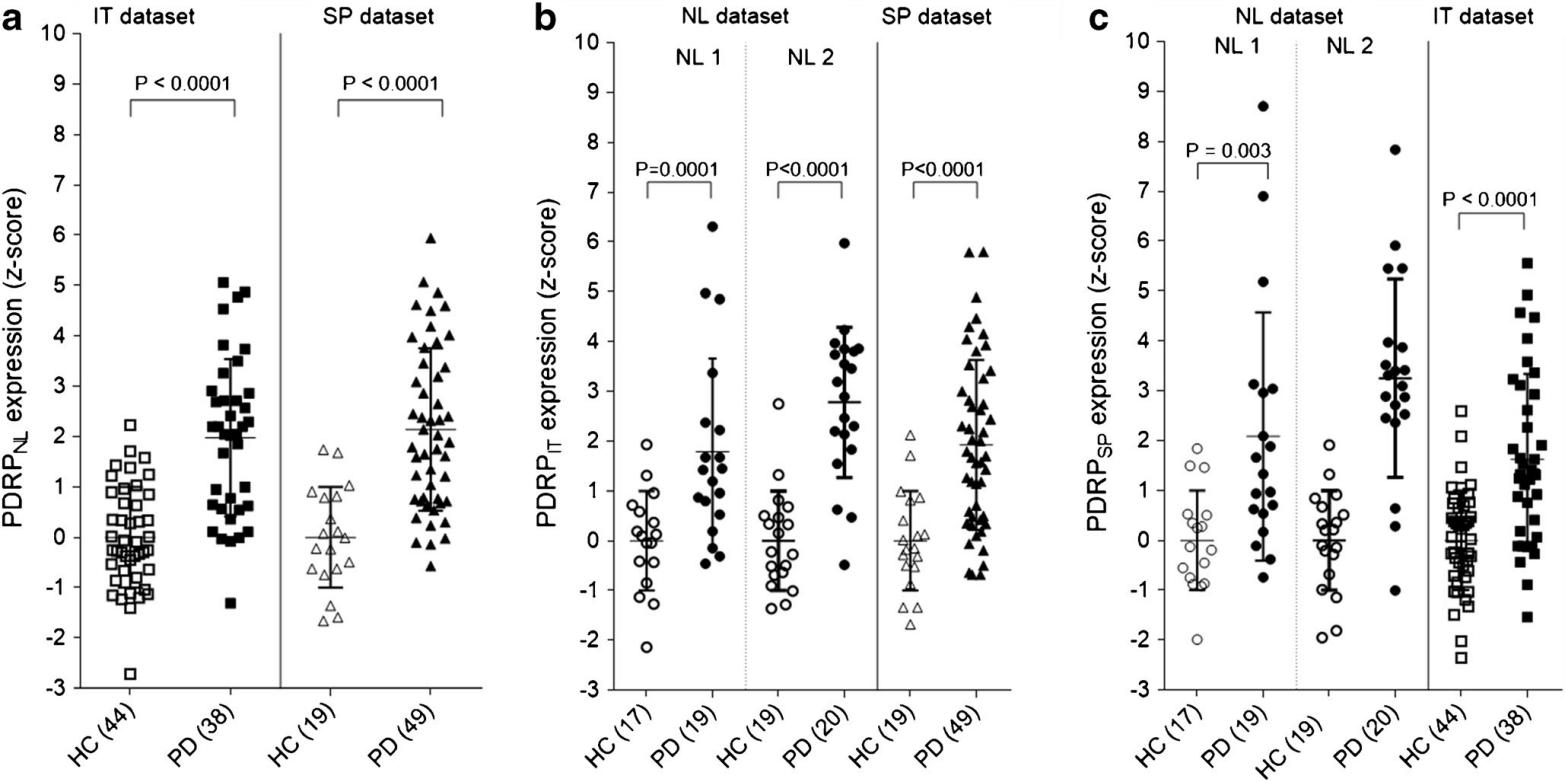
Subject scores for each PDRP in the other cohorts (cross-validation). **a** PDRP_NL_ subject scores are plotted for the Italian (IT) and Spanish (SP) data. **b** PDRP_IT_ subject scores are plotted for the two Dutch samples (NL1 and NL2) and in SP data. **c** PDRP_SP_ subject scores are plotted for NL1, NL2, and IT data. Subject scores are *z*-transformed to healthy control values from the same camera, and compared between groups with a Student’s *t* test. Bars indicate mean and standard deviation

**Fig. 5:**
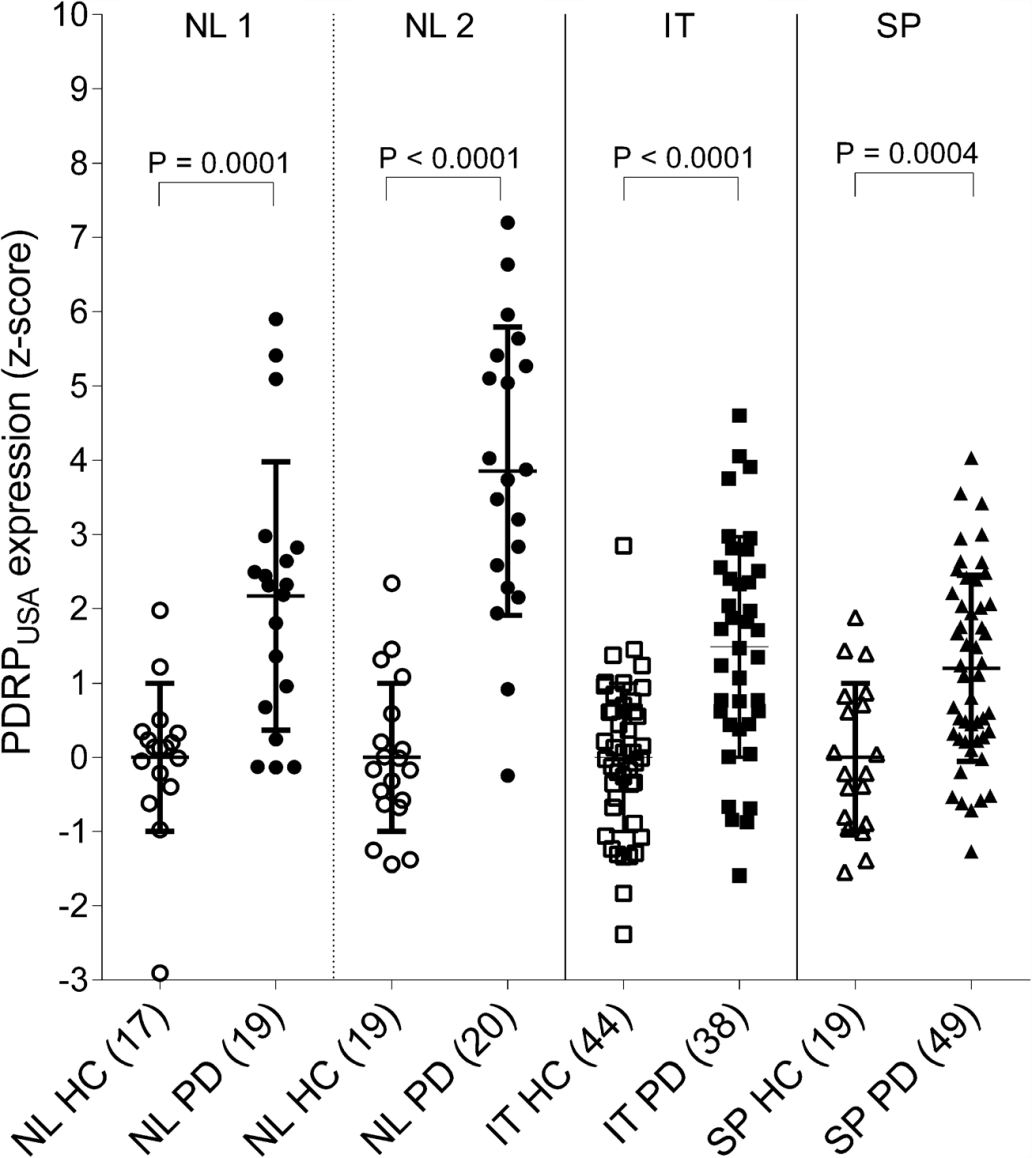
Subject *z*-scores for the reference pattern PDRP_USA_ [11] in each of the datasets. Subject scores are *z*-transformed to healthy control values from the same camera, and compared between groups with a Student’s *t* test. Bars indicate mean and standard deviation

Each of the PDRPs (including the PDRP_USA_) was significantly expressed in PD patients compared with controls, in each of the datasets (Figs. 4a–c and 5). Corresponding ROC-AUCs are reported in Table 4.

**Table 4:** Cross-validation of patterns

	NL dataset 1	NL dataset 2	IT dataset	SP dataset
N HC/PD	17/19	19/20	44/38	19/49
PDRP_NL_ AUC		0.96	0.86	0.87
PDRP_IT_ AUC	0.81	0.93	0.83^†^	0.83
PDRP_SP_ AUC	0.82	0.92	0.80	
PDRP_USA_ AUC	0.85	0.95	0.79	0.76

Subject scores for each PDRP were obtained in each dataset and subsequently *z*-transformed (see Figs. 3 and 4). With these scores, a receiver operating curve was plotted (for each pattern in each dataset) and the area under the curve (AUC) was obtained

^†^Obtained from the IT validation cohort (HC = 24; PD = 18)

Correlations to UPDRS and disease duration were inconsistent (Table 5). Within each dataset, *z*-scores of any two PDRPs were significantly correlated. Subject scores on all three patterns were also significantly correlated to subject scores on PDRP_USA_ (Table 5). Especially, the PDRP_NL_ showed consistent high correlations to PDRP_USA_. In addition, a comparison between spatial topographies of the original PDRP_USA_ versus the PDRP_IT_, PDRP_NL_, and PDRP_SP_ showed significant correlations in region weights (Table 6).

**Table 5:** Correlations between PDRP subject scores and clinical data

NL data								
	Age (HC)	Age (PD)	Disease duration	UPDRS (off)	PDRP_NL_	PDRP_IT_	PDRP_SP_	PDRP_USA_
NL1								
PDRP_IT_	− 0.02	0.24	0.50*	0.38			0.84***	0.79***
PDRP_SP_	0.16	0.20	0.50*	0.42		0.84***		0.71***
PDRP_USA_	0.64**	0.50*	0.60**	0.49^*^		0.79***	0.71***	
NL2								
PDRP_NL_	0.20	0.590**	0.087	NA		0.89***	0.76***	0.93***
PDRP_IT_	0.07	0.387	0.229	NA	0.89***		0.87***	0.75***
PDRP_SP_	0.13	0.459*	0.102	NA	0.76***	0.87***		0.72***
PDRP_USA_	0.46*	0.698**	0.070	NA	0.93***	0.75***	0.72***	
IT data								
PDRP_NL_	0.30	0.48**	0.04	0.35*		0.87***^†^	0.73***	0.92***
PDRP_IT_	0.34^†^	0.23^†^	− 0.05^†^	0.44^†^	0.87***^†^		0.78***^†^	0.68***^†^
PDRP_SP_	0.46**	0.41*	− 0.20	0.47**	0.73***	0.78***^†^		0.78***
PDRP_USA_	0.43**	0.48**	− 0.05	0.33*	0.92***	0.92***^†^	0.78***	
SP data								
	Age (HC)	Age (PD)	Disease duration	UPDRS (on)	PDRP_NL_	PDRP_IT_	PDRP_SP_	PDRP_USA_
PDRP_NL_	0.03	0.33*	0.26	− 0.01		0.91***	0.81***^†^	0.92***
PDRP_IT_	− 0.02	0.21	0.25	− 0.01	0.91***		0.77***^†^	0.82***
PDRP_SP_		0.33^†^	0.43*^††^	0.01^††^	0.81***^††^	0.77***^††^		0.84***^††^
PDRP_USA_	− 0.11	0.34*	0.21	− 0.09	0.92***	0.82***	0.84***^†^	

*Significant at *P* < 0.05; **Significant at *P* < 0.01; ***Significant at *P* < 0.001

*NA* not available

^†^Obtained from the IT validation cohort (HC = 24; PD = 18)

^††^Obtained from the SP validation cohort (PD = 30)

**Table 6:** Region-weight correlations

	PDRP_USA_	PDRP_IT_	PDRP_NL_	PDRP_SP_
PDRP_USA_		0.67***	0.78***	0.481**
PDRP_IT_	0.67***		0.68***	0.304
PDRP_NL_	0.78***	0.68***		0.458*
PDRP_SP_	0.48**	0.30	0.458*	

*Significant at *P* < 0.05; **Significant at *P* < 0.01; ***Significant at *P* < 0.001

### PDRP subject scores in MSA-p patients

**Fig. 6:**
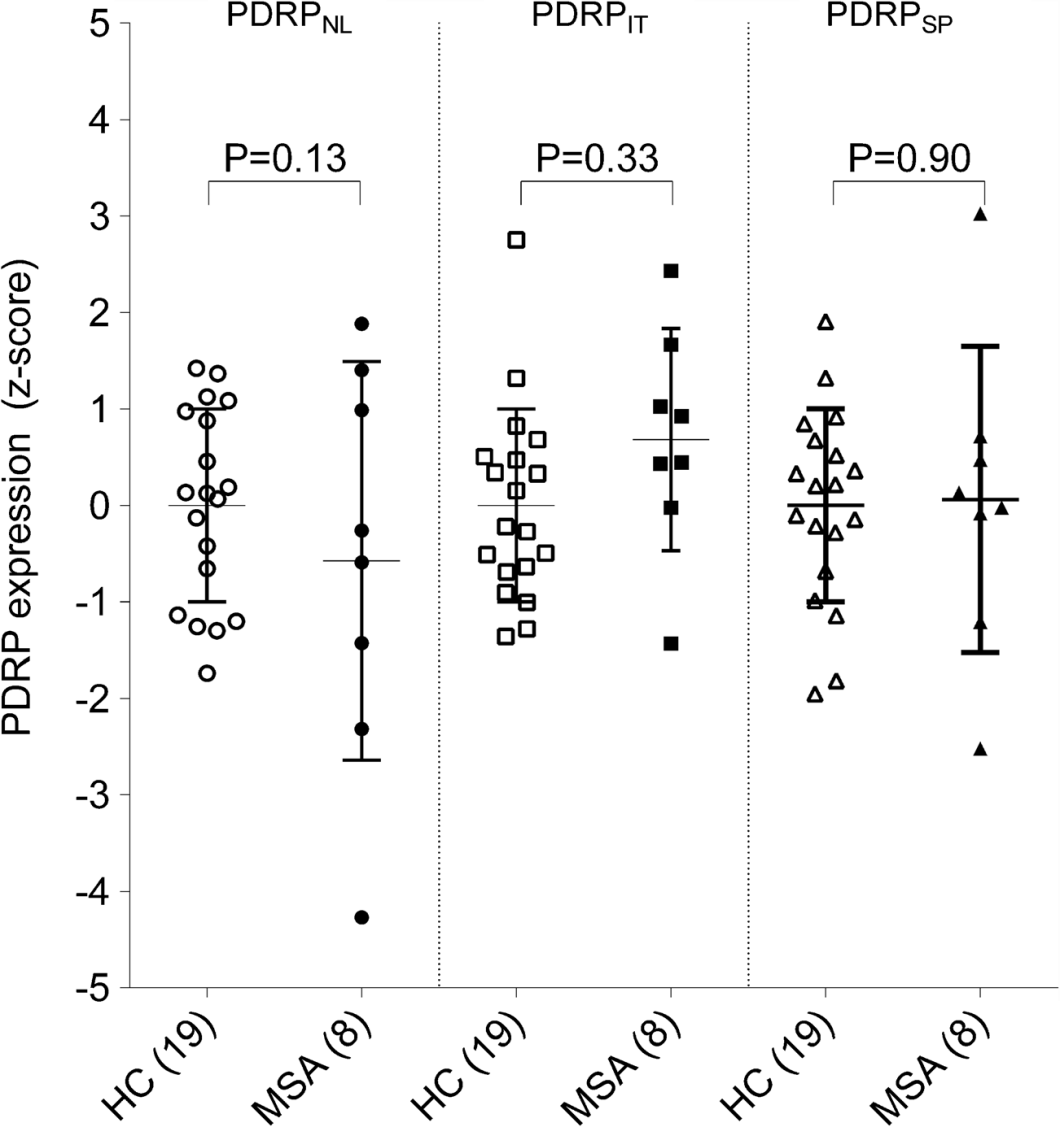
Subject scores for each PDRP in eight cases of MSA-p. Subject scores are *z*-transformed to NL2 controls and compared between groups with a Student’s *t* test. Bars indicate mean and standard deviation

Subject scores for each PDRP were calculated in MSA patients. Subject *z*-scores on each PDRP were not significantly different between controls and MSA patients (Fig. 6).

### Principal component 1

As stated above, PDRP_NL_ and PDRP_IT_ were identified as linear combinations of multiple PCs. All analyses were repeated for PDRP_NL_-PC1, PDP_IT_-PC1, and PDRP_SP_-PC1. The PDRPs that were based on combinations of PCs yielded higher diagnostic accuracy (Table 4) compared with patterns based on PC1 alone (Table 7). However, subject scores on PDP_IT_-PC1, PDRP_NL_-PC1, and PDRP_SP_-PC1 did show much higher correlations to subject scores on PDRP_USA_ (all *r* > 0.98, *P* < 0.0001).

**Table 7:** Receiver operating curve—AUCs using PC1

	NL dataset 1	NL dataset 2	IT dataset	SP dataset
HC/PD	17/19	19/20	44/38	19/49
PDRP_NL-PC1_ AUC		0.92	0.77	0.78
PDRP_IT-PC1_ AUC	0.78	0.95	0.81^†^	0.72
PDRP_SP-PC1_ AUC	0.84	0.96	0.77	

^†^Obtained from the IT test cohort (HC = 24; PD = 18)

## Discussion

In this study, we cross-validated the previously published PDRP_NL_ [24], and additionally identified a PDRP in an Italian (PDRP_IT_) and Spanish (PDRP_SP_) sample. The three patterns were akin to PDRP_USA_, and also to the PDRP described in other populations [20, 21]. Topographical similarity to PDRP_USA_ was confirmed for each of the three PDRPs by a significant correlation of region weights, and a significant correlation in subject scores. Furthermore, PDRP_NL_, PDRP_IT_, and PDRP_SP_ were significantly expressed in PD patients compared with controls in both identification and validation cohorts, but were not significantly expressed in MSA-p patients.

The typical PDRP topography is characterized by relative hypermetabolism in the thalamus, putamen/pallidum, pons, cerebellum, and motor cortex. These changes co-vary with relatively decreased metabolism in the prefrontal, parietal, temporal, and occipital cortices [11, 15, 20, 21, 23, 24]. This topography is thought to reflect changes in cortico-striatopallido-thalamocortical (CSPTC) loops and related pathways in PD [33, 34]. In these circuits, a dopaminergic deficit leads to abnormal basal ganglia output, mediated by hyperactivity of the subthalamic nucleus (STN) and its efferent projections. Several studies support a direct relationship between altered STN output and the PDRP topography [16, 35–38].

The high degree of similarity in PDRP topography across samples is striking considering differences in demographics, clinical characteristics, scanning methods, and reconstruction algorithms. Especially the PDRP_NL_ was highly similar to the reference pattern (PDRP_USA_). These two patterns showed the highest subject score correlation and region weight correlation. Furthermore, the PDRP_NL_ achieved the highest AUC in the other cohorts. Like PDRP_USA_, PDRP_NL_ was derived in a cohort of off-state patients with a wide range of disease durations (duration 4.4 ± 3.2 years; range 1.5–11.5 years) and severities.

Deviations from the typical PDRP topography are worth exploring further in relation to clinical characteristics. The PDRP_IT_ is unique in that it is, to our knowledge, the first time the PDRP has been derived in “de-novo,” treatment-naïve PD patients. It is likely that these very early-stage patients have a less severe nigrostriatal dopaminergic deficit compared with the more advanced PD patients in PDRP_USA_, PDRP_NL_, and PDRP_SP_ derivation cohorts. This may be reflected by less severe involvement of the frontal cortex in PDRP_IT_, as nigrostriatal denervation is known to be positively correlated with hypometabolism in the frontal cortex [13, 39].

The PDRP_SP_ was derived in PD patients who were scanned while being on dopaminergic medication. Levodopa is known to decrease metabolism in the cerebellar vermis, putamen/pallidum, and sensorimotor cortex. Levodopa therapy can reduce PDRP expression, but does not completely correct the underlying network abnormalities [16]. It can be assumed that the effect of dopaminergic therapy on PDRP expression is modest in comparison with the effect of disease progression [40]. Indeed, the typical PDRP topography could still be identified in these *on*-state patients. However, the PDRP_SP_ did not correlate as well to the other patterns, both in terms of subject scores and region weights. It is not clear whether this is related to the advanced disease stage or the effect of treatment. The PDRP_SP_ was characterized by more stable involvement of the occipital cortex, possibly related to the presence of mild cognitive impairment and visual hallucinations, which often occur in advanced PD [41].

Following from the above, it can be concluded that the typical PDRP topography is highly reproducible. Similar topographies have also been identified in studies comparing ^18^F-FDG-PET scans of healthy controls and PD patients with SPM [3–7]. Such analyses can be supportive in the visual assessment of an ^18^F-FDG-PET scan in clinical practice. Several studies have evaluated the diagnostic value of observer-dependent visual reads supported by SPM-based comparisons to healthy controls [3, 4, 42–44]. A recent meta-analysis (PD versus “atypical” parkinsonism) estimated a pooled sensitivity of 91.4% and specificity of 94.7% for this semi-quantitative approach [45].

The merit of SSM PCA over mass-univariate approaches lies in its ability to objectively quantify ^18^F-FDG PET scans of patients using the pre-defined patterns. Pattern expression scores were shown useful in differential diagnosis, tracking disease progression, and estimating treatment effects [46]. Although in the current study PDRP *z*-scores were significantly higher in PD patients compared with healthy controls, there was a considerable overlap in PDRP *z*-scores between patients and controls in almost every cohort. This overlap is not unique to the current data, and is also apparent in other studies [12].

Some healthy controls appear to express the PDRP. Since we found significant correlations between PDRP *z*-scores and age in healthy controls, it could be suggested that ageing and PD share certain metabolic features. Metabolic decreases have been reported in the parietal cortex in normal aging [47, 48]. This may cause some overlap with the PDRP. However, the correlation with age in our study was not consistent across all datasets and patterns (Table 5). Furthermore, expression of an age-related spatial covariance pattern was shown to be independent from PDRP expression [49, 50]. Alternatively, a high PDRP *z*-score in a healthy subject could signal a prodromal stage of neurodegeneration. For instance, subjects with idiopathic REM sleep behavior disorder (a prodromal stage of PD) were shown to express the PDRP years before onset of clinical parkinsonism [17, 25].

Low PDRP *z*-scores in PD patients could indicate inaccurate clinical diagnosis. A recent meta-analysis of clinicopathologic studies suggests that the clinical diagnosis of PD by an expert, after an adequate follow-up, has a sensitivity of 81.3% and a specificity of 83.5% [51]. Thus, even under ideal circumstances, the diagnosis is inaccurate in a number of patients.

Overlap in pattern expression scores is not only apparent between controls and PD patients, but also between patients with different parkinsonian disorders. For instance, the PDRP may also be expressed in patients with progressive supranuclear palsy (PSP) [52]. This means that the expression score for a single disease-related pattern is inadequate to differentiate between multiple disorders. However, this does not hamper application in differential diagnosis. Previous studies have shown that an algorithm combining multiple disease-related patterns (including the PDRP) with logistic regression could accurately distinguish between parkinsonian disorders. With this method, Tang et al*.* achieved accurate categorization of patients (*n* = 167) with an uncertain diagnosis 3–4 years before a final clinical diagnosis was made by an expert clinician masked to the imaging findings [18]. Highly similar results were obtained in an independent sample (*n* = 129) [19].

In this study, we compared data from different centers. It is well-known that variations in PET scanners and image reconstruction algorithms influence disease-related pattern scores [53–55] (supplementary Fig 1). In support of this, we recently identified clear center-specific features in the current data using machine-learning algorithms [56]. Therefore, PDRP subject scores cannot be compared readily between subjects from different centers. In all PDRP studies, this is solved with a *z*-transformation using the mean and standard deviation of a small healthy control group. This potentially introduces a bias, depending on which controls are selected. However, this issue is not relevant for *within* subject studies. Therefore, PDRP subject scores may be especially useful in tracking disease progression [14], or treatment effects [16, 35–38].

This study is methodologically different from previous PDRP studies. The PDRPs identified in this study were formed by a combination of principal components (PCs). These combinations were determined based on a forward stepwise logistic regression model [30]. There are different methods to decide which components are included in the disease-related pattern [10]. Previous studies have always identified the PDRP as PC1 in isolation [11, 20, 21]. The process of component selection is not always described in detail. Automatically choosing PC1 as the disease-related pattern, and disregarding consecutive, smaller PCs, increases the risk information loss. On the other hand, a pattern that combines multiple PCs may give a better fit of the initial sample, but may be limited in its relevance or generality across new datasets. Therefore, we re-evaluated the data and included only PC1 for PDRP_IT_, PDRP_NL_, and PDRP_SP_. Indeed, the PC1 patterns correlated better to the reference pattern (PDRP_USA_). However, the patterns that included multiple PCs yielded higher diagnostic accuracy . Apart from component selection, several other decisions and cutoffs may influence pattern identification [10]. More advanced machine-learning algorithms may be of use in determining optimal patterns without the use of arbitrary thresholds and associated loss of potentially useful information [55–58].

There is increasing interest to apply the PDRP in clinical practice and in therapeutic trials [12]. However, rigorous validation by independent research groups is necessary before widespread application. The current study has contributed to the finding that the PDRP is a universal feature of PD, and it is striking that such similar patterns could be identified in a limited number of ^18^F-FDG PET scans from three populations, despite overt clinical and methodological heterogeneity. However, our results also show considerable overlap in PDRP subject scores between control and PD groups. Further study is needed to overcome this issue, perhaps by addressing potential center-specific effects in the data or by employing more advanced machine-learning algorithms.

## Electronic supplementary material

ESM 1 (DOCX 62 kb).
