# Supplementary material for: Abnormal pattern of brain glucose metabolism in Parkinson’s disease: replication in three European cohorts
Source: Eur J Nucl Med Mol Imaging. 2019 Nov 25;47(2):437–50. doi: 10.1007/s00259-019-04570-7 (PMC6974499; doi:10.1007/s00259-019-04570-7)
Supplement: Supplementary file 1 — (DOCX 62 kb). [file 259_2019_4570_MOESM1_ESM.docx]

**Supplementary Fig 1**: PDRP_NL_ subject scores (not z-transformed) are plotted for NL1 and NL2 controls. The difference in mean ‘raw’ PDRP_NL_ score between NL1 and NL2 controls is tested for significance with a student’s T-test.

**
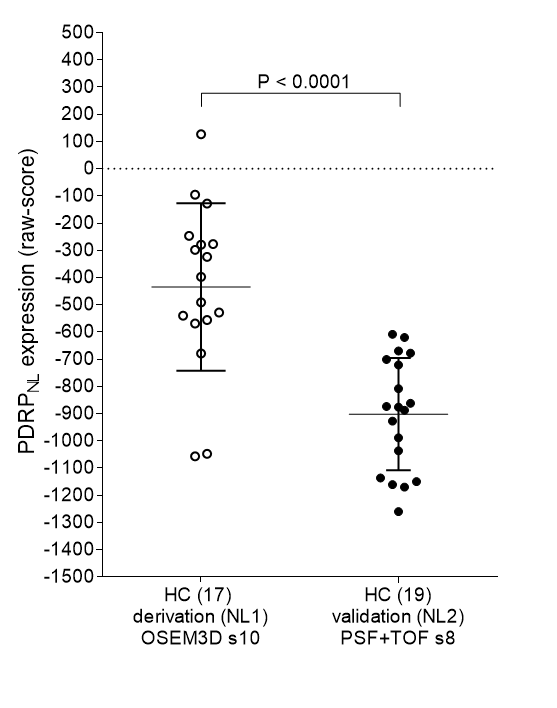
**
